# Alumni of Harvard Dental School

**Published:** 1900-10

**Authors:** 


### ALUMNI OF HARVARD DENTAL SCHOOL.

Dr. Edward C. Briggs read a paper entitled "Some of the Nerve Remedies."

(For Dr. Briggs's paper, see page 659.)

### DISCUSSION.

*Dr. Gillett.*—I have used some of the drugs Dr. Briggs has mentioned, and have sometimes been helped over hard places by the use of the coal-tar products.

Antikamnia and ammonol are often serviceable in quieting the patient who must go through severe suffering with abscesses or other inflammatory conditions. I think antikamnia has served me better than the ammonol.

Orthoform, a comparatively new local anæsthetic, has served me well in some cases for relieving pain after operations. The orthoform powder dusted over the surface of a wound will often give relief from pain for twenty-four to forty-eight hours. After the extraction of third molars, where there has been laceration and bruising of the tissues, it is a very satisfactory application. Orthoform dissolves very slowly, which fact probably accounts for its prolonged anææsthetic effect.

Nervanin is closely related to orthoform, and is intended for hypodermic use, but I have been waiting for more extended reports before making use of it.

*Dr. Kelley.*—I have used orthoform very successfully. I have also had a great deal of experience with antikamnia, and have great faith in it, although I seem to get very poor results from it. In the terrible pain which occurs after the extraction of teeth, I find orthoform placed in the socket very successful.

I would like information in regard to an extremely unfortunate case which I have at present. A young lady, about twenty-two or twenty-three years of age, has lost within the last year and a half seventeen teeth, sixteen before she came under my care. All were extracted on account of extreme pain. She came to me with the seventeenth, and I have lost that. The pain seemed to start with a tooth so nearly perfect that I could not easily discover anything the matter with it; only after a very careful examination I discovered a small cavity which had been filled with gutta-percha. She has had a very skilful dentist, but nothing seems to give relief.

The trouble goes from the right side to the left and from the upper to the lower jaw; from the back of the mouth to the front. She has not lost any of her front teeth, but she constantly fears that possibility. I decided, after an examination of the tooth under my care, to destroy the pulp. I filled the root-canal, but as soon as my instrument went through the apical foramen a terrible pain started up. This continued steadily, and all the drugs just mentioned could not stop it. I do not remember just what I used, but I should say about everything, and I had, of course, taken out my root-filling. She went home and to bed, and three or four days afterwards her family physician extracted the tooth.

*Dr. Briggs.*—You did not see the tooth when it came out?

*Dr. Kelley.*—Yes, I saw the tooth. There was a little inflammation of the pericementum. It had been perfectly cleaned out, root perfectly filled down to the apex, and everything looked all right.

*Dr. Briggs.*—Did you see whether the pain was caused by the formation of pulp-stone or decalcifying of the teeth? This pain is due to the presence of the pulp, and as long as the patient's general health is depreciated, such conditions are liable to occur. I should be ready to spring very quickly to any tooth that showed any signs of decay, destroy the pulp, and fill it immediately. I should be very quick to act the minute any tooth was suspected of decay.

*Dr. Kelley.*—The pain seemed to start the moment I got down to the end of the root.

*Dr. Briggs.*—Whatever the condition was, you simply started up the seat of the trouble. Probably if you could have controlled that one attack, you would not have had another one.

*Dr. Kelley.*—I thought possibly the girl could not stand the pain resulting from the root-filling, although she seemed to be very courageous, and she certainly was called upon to stand a great deal. However, I feel it is the loss of physical and nerve force that is more the cause of unsuccessful treatment than any other one thing. It seems to be a case where the patient is panic-stricken.

